# Bearing Fruit: Miocene Apes and Rosaceous Fruit Evolution

**DOI:** 10.1007/s13752-022-00413-1

**Published:** 2023-01-04

**Authors:** Robert N. Spengler, Frank Kienast, Patrick Roberts, Nicole Boivin, David R. Begun, Kseniia Ashastina, Michael Petraglia

**Affiliations:** 1 Department of Archaeology, Max Planck Institute for Geoanthropology, Jena, Germany; 2 Domestication and Anthropogenic Evolution Research Group, Max Planck Institute for Geoanthropology, Jena, Germany; 3 Senckenberg Research Station of Quaternary, Palaeontology, Weimar, Germany; 4 isoTROPIC Research Group, Max Planck Institute for Geoanthropology, Jena, Germany; 5 Department of Anthropology, National Museum of Natural History, Smithsonian Institution, Washington, DC USA; 6 School of Social Science, The University of Queensland, Brisbane, Australia; 7 Department of Anthropology and Archaeology, University of Calgary, Calgary, Canada; 8 Department of Anthropology, University of Toronto, Toronto, Canada; 9 Australian Research Centre for Human Evolution, Griffith University, Nathan, Queensland Australia

**Keywords:** Apes, Megafauna, Miocene, Mutualism, Rosaceae, Seed dispersal

## Abstract

Extinct megafaunal mammals in the Americas are often linked to seed-dispersal mutualisms with large-fruiting tree species, but large-fruiting species in Europe and Asia have received far less attention. Several species of arboreal Maloideae (apples and pears) and Prunoideae (plums and peaches) evolved large fruits starting around nine million years ago, primarily in Eurasia. As evolutionary adaptations for seed dispersal by animals, the size, high sugar content, and bright colorful visual displays of ripeness suggest that mutualism with megafaunal mammals facilitated the evolutionary change. There has been little discussion as to which animals were likely candidate(s) on the late Miocene landscape of Eurasia. We argue that several possible dispersers could have consumed the large fruits, with endozoochoric dispersal usually relying on guilds of species. During the Pleistocene and Holocene, the dispersal guild likely included ursids, equids, and elephantids. During the late Miocene, large primates were likely also among the members of this guild, and the potential of a long-held mutualism between the ape and apple clades merits further discussion. If primates were a driving factor in the evolution of this large-fruit seed-dispersal system, it would represent an example of seed-dispersal-based mutualism with hominids millions of years prior to crop domestication or the development of cultural practices, such as farming.

## Introduction

**Fig. 1:**
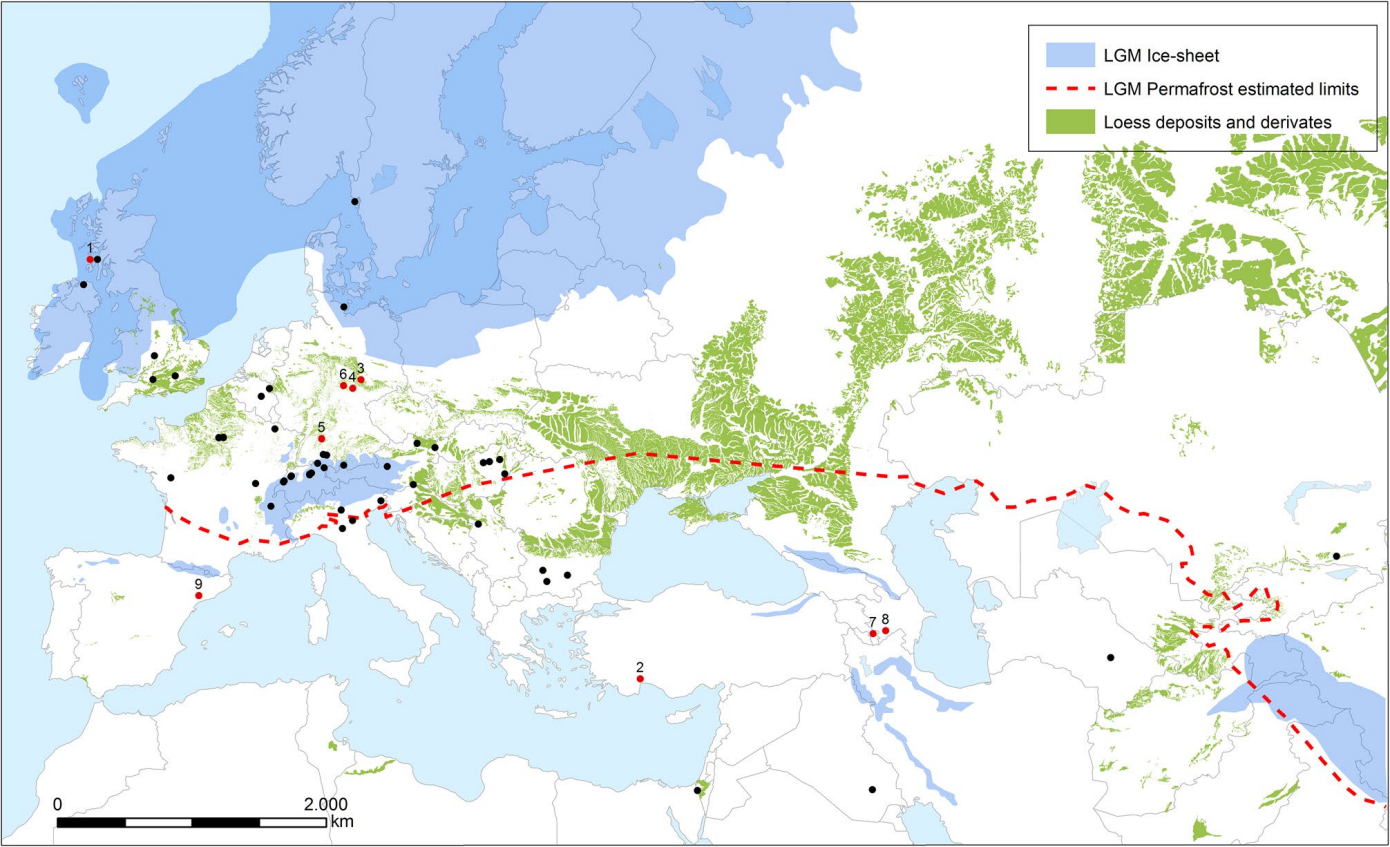
Map showing locations of archaeobotanical finds of apple seeds during the mid-Holocene in black from Spengler (2019); red data points represent Pleistocene sites with apples and humans or artifacts found at the same site: (1) Staosnaig, Scotland, Mesolithic (Carruthers 2000; Mithen et al. 2001); (2) Öküzini, Turkey, Epipaleolithic (Martinoli and Jacomet 2004); (3) Neumark Nord, Germany, Middle Pleistocene (Gregor and Vodickova 1983; Schweigert 1991); (4) Ehringsdorf, Germany, 243–200 ka (Vent 1974; Schwarcz et al. 1988; Mallick and Frank 2002); (5) Stuttgart-Untertürkheim, Germany, Pleistocene (Mai 2010); (6) Burgtonna, Germany, 130–115 ka (Vent 1978); (7) Shamb outcropping, Armenia, 53 ka (Ollivier et al. 2010); (8) Azokh 1 Cave, Armenia, 184–100 ka (Allué 2016); and (9) Molí del Salt, Spain, 11,000–12,500 (Allué 2016). Most of the data points fall in Pleistocene forest refugial zones, suggesting that these large-fruiting clades did not colonize new areas during the early Holocene, likely due to a lack of dispersers. Areas of loess deposits modified from Börker et al. (2018) and Bateman and Catt (2007)

The history of the domesticated apple (*Malus pumila* (formerly *M. domestica*)) is closely intertwined with humans. There are 3,000 species in the Rosaceae family, many of which are economically important, such as apples, peaches (*Prunus persica*), plums (*Prunus* spp.), and pears (*Pyrus* spp.). *Malus* encompasses 55 species (Hancock et al. 2008), of which the apple is the most morphologically diverse, with over 10,000 landraces of apples recognized worldwide (Sau et al. 2018). Human-facilitated gene flow has caused major changes in population genetics within *Malus* over the past few centuries, leading to immense phenotypic change in fruit structure (Urrestarazu et al. 2016). Recent archaeobotanical and genetic research suggests that the modern apple evolved through hybridization caused by human-induced movement of trees between isolated populations during the late Holocene (Harris et al. 2002; Gross et al. 2013; Cornille et al. 2015). The geographically restricted populations of some large-fruiting *Malus* trees, such as *M. orientalis* and *M. sieversii*, appear today to map over former glacial refugia (Fig. 1) (Spengler 2019). Humans started spreading the seeds beyond these refugial pockets in association with the Neolithization of Europe and the development of transcontinental exchange networks (Cornille et al. 2015; Duan et al. 2017; Spengler 2019). Although this period represented a significant evolutionary change for these plants as they responded to human dispersal and maintenance of the population, the appearance of large sugary fruits actually occurred roughly nine million years ago (Ma) and their interactions with hominid populations may also have a far greater time depth.

Megafruits (defined as any fruit too large for typical avian dispersal, > 25 mm) evolved in parallel at least twice (Prunoideae and Maloideae) across the Rosaceae clade in Eurasia starting in the late Miocene (Xiang et al. 2017). Prominent examples of Maloideae megafruits with highly restricted ranges in the wild include *Cydonia* spp., *Pyrus* spp., *Malus* spp., *Mespilus germanica*, *Sorbus domestica*, and *Eriobotrya japonica*. This leaves open the question as to which seed dispersers the trees originally evolved larger fruits to recruit. It is clear that the mutualistic relationship between humans and apples stretches much further back than the Neolithic. Heavy foraging of wild European apples by pre-farming peoples undoubtedly manipulated gene flow and population dynamics. Some scholars have suggested that these pre-agricultural foragers were directly maintaining apple trees (Clarke 1978). Intentional burning would have increased forest patchiness, facilitating *Malus* sp. growth and dispersal (Zvelebil 1994; Kaplan et al. 2016). In certain parts of Central Europe, wild apples were an important part of the Neolithic economy, and foragers may have been targeting remnants of refugial apple populations (Antolín et al. 2016). Wild apple foraging was practiced during the Pleistocene; *Malus* seeds have been recovered from the Staosnaig site on the Isle of Colonsay, Scotland (Carruthers 2000; Mithen et al. 2001), and large-fruiting Rosaceae seeds are recorded as foraged foods across Mesolithic Europe (Zvelebil 1994). In addition, seeds of wild pears (*Pyrus pyraster*) were recovered from the Epipaleolithic cave site of Öküzini in Anatolia, along with other wild arboreal fruit seeds (Fig. 1) (Martinoli and Jacomet 2004).

**Fig. 2:**
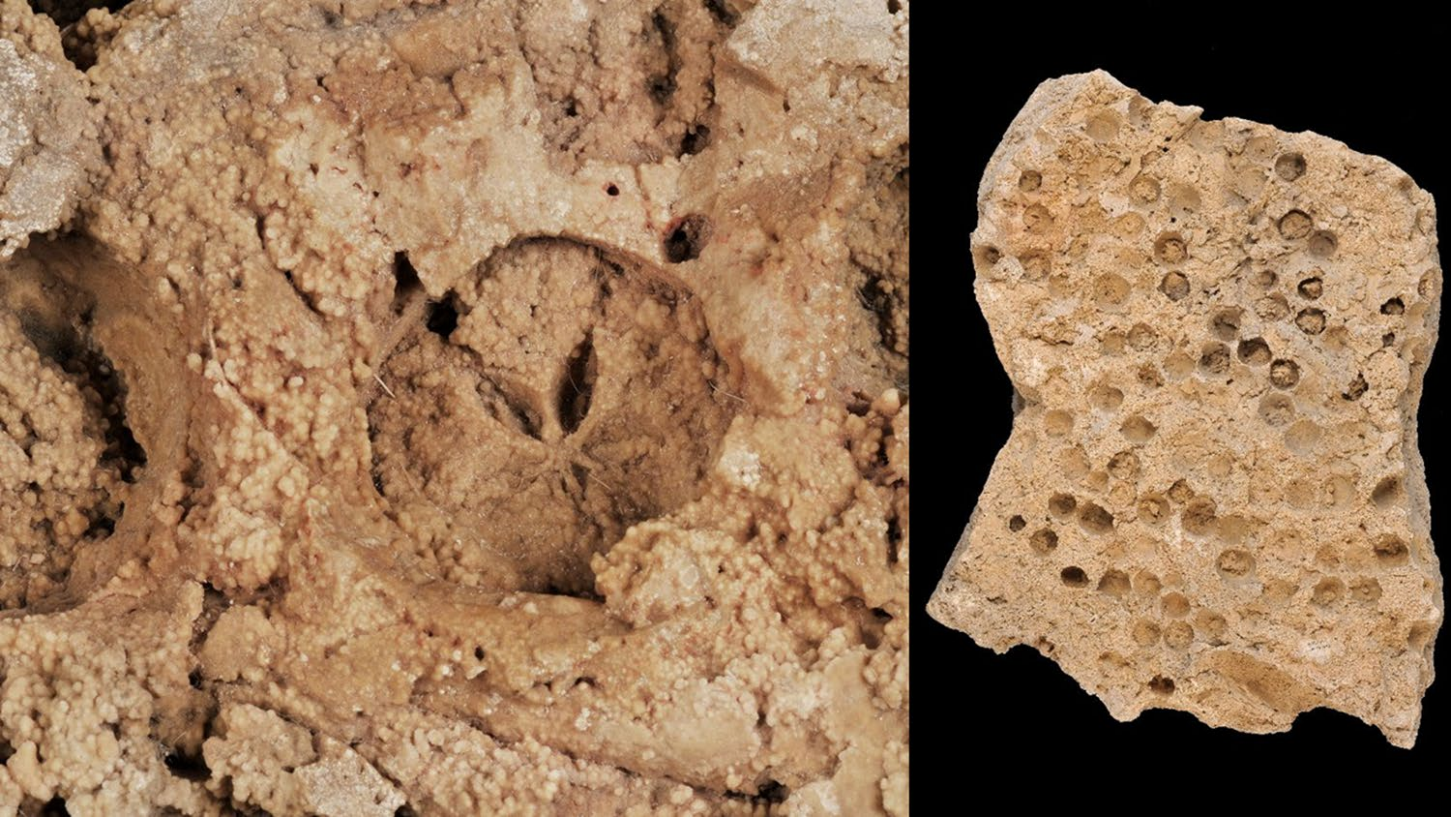
Images of fossil apple impressions from the ca. 243,000–200,000-year-old travertine site of Ehringsdorf, Germany. This fossil represents one of the earliest direct linkages of hominins and large-fruiting *Malus* sp.

Hominin collection and use of these fruits appears to extend even beyond the appearance of our own species in Eurasia, hinting at a much deeper time depth for human/*Malus* seed-dispersal mutualism. For example, fossil apple impressions in travertine from the Ehringsdorf site (Fig. 2) of central Germany (Vent 1974) place large-fruiting apples in Central Europe between 243,000 and 200,000 years ago during Marine Isotope Stage 7 (Schwarcz et al. 1988; Mallick and Frank 2002). The fossils at Ehringsdorf include several hominin remains, characterized either as early Neanderthal, pre-Neanderthal (Vlcek 1993), or typical Neanderthal with Mousterian stone tools (Hublin 2009). The site contains several occupations, including hearths, preserved in rapidly hardening travertine and provides some of the earliest linkages between *Homo* and apples (Behm-Blancke 1960; Kot 2017). Apple fossils have also been recovered at the middle Pleistocene interglacial site of Neumark Nord (Geiseltal, Germany) (Mai 2010). Resembling the travertine incrustations from Ehringsdorf (Fig. 2), numerous apple impressions were also preserved in travertine deposits at Stuttgart-Untertürkheim, which were presumed to be gathered by hominins (Gregor and Vodickova 1983; Schweigert 1991).

There are also further, more tentative, deep time links between hominins and Maloideae. Although the identification of apples from leaves was also originally made at Burgtonna, in Eemian travertine of Germany (Vent 1978), this identification has been refuted as *Lonicera arborea* by Schweigert (Schweigert 1991). Other Pleistocene sites in refugial zones of Europe have provided evidence of large-fruiting Rosaceae, including *Mespilus germanica* which was also reported at Burgtonna (Vent 1978). Travertine formations dating to both the late Pleistocene (53,000 BP) and early Holocene (12,000 BP) in the Shamb outcropping of southern Armenia, a former forest refugium, contain leaf impressions from *Malus* sp., *Prunus* sp., and *Pyrus* sp. (Ollivier et al. 2010). In the same Pleistocene forest refugia, wood charcoal analyses from Azokh 1 Cave in Armenia (184,000–100,000 years ago), illustrate that 80% of the wood that hominins (both Neanderthal and modern humans are present in varying layers) were burning was from *Prunus* sp.; among the remaining 20% of charcoal were fragments of Maloideae and other fruit trees, such as *Paliurus/Ziziphus* and *Celtis/Zelkova* (Allué 2016). Similarly, Maloideae wood was found with *Prunus* sp. wood fragments and a carbonized seed of *Prunus spinosa* at the site of Molí del Salt in northeastern Iberia (Allué et al. 2010).

These publications suggest: (1) wild fruit forests were common in pre-Holocene Eurasia; (2) many of the megafruits with restricted ranges during the mid-Holocene (e.g., *Mespilus*) may have had much wider ranges across refugial pockets during the Pleistocene; and (3) humans were likely utilizing these resources. In this article, we take this evidence for hominin–*Malus* spp. interactions a stage further to argue that the evolution of large fruits in the Rosaceae lineage may have been linked to seed-dispersal-based mutualism prior to the Pleistocene. We base our hypothesis on: (1) the fossil and genetic record for *Malus* spp.; (2) morphological traits of fruits in the clade; (3) the dominance of frugivorous or folivorous primates on the landscape during the development of fleshy fruits in the Miocene; (4) the fossil and extant record for large primate frugivory; and (5) the prominence of seed dispersal in other primate clades.

## Seed-Dispersal-Based Mutualisms

Large rosaceous fruits evolved to recruit animals on a different landscape than that of modern Eurasia. Continual dispersal through the Pleistocene and Holocene was likely facilitated by ursids, equids, and elephantids. Bears have long been theorized as one of the dispersers of the Tian Shan wild apples, and we emphasize the likelihood that bears have been a key disperser for these fruits through the Holocene. Personal observations by the lead author attest to the germination of apple seeds after digestion by North American black bears. Many studies have emphasized the success of bears as seed dispersers, although, in these cases, largely for small-fruiting plants (García‑Rodríguez et al. 2021).

Ecologists tend to agree that plants with small fleshy fruits largely evolved for avian dispersal (Tiffney 2004; Sussman et al. 2013), but, with the influx of African frugivorous megafauna in the early Miocene, including large primates and certain groups of proboscideans and perissodactyls, some angiosperms found more effective dispersal mechanisms (Van der Made and Mazo 2003; Begun et al. 2012). European megafaunal mammals prior to the early Miocene migrations were largely adapted for grazing (Steininger et al. 1985; Van der Made 1999). The new megafaunal dispersers that migrated to Europe in the late Miocene, by contrast, would have been able to spread much larger seeds than their avian or small mammalian predecessors. The spread of African frugivorous species into Europe was coupled with a climatic shift or a series of climatic changes over millions of years (Van der Made and Mazo 2003). During the Miocene, in mid-latitudes, north of the Tropic of Cancer, climates fluctuated wildly from much hotter and more humid than today to much drier. The late Miocene saw an encroachment of drier, more open deciduous forests and advances of grassland biomes, and was the key period for the evolution of large-fruiting rosaceous trees and shrubs (Mai 1995; Xiang et al. 2017). *M. sieversii* trees evolved for an open steppe or savanna landscape and only grow to two to ten meters tall; like most large-fruiting Rosaceae trees, they cannot survive under a forest canopy. This growth habit mandates a long-distance seed-dispersal strategy to ensure directed colonization to suitable gaps in the forest cover. Biotic dispersal often leads to directed dispersal, targeting prime colonization areas (Eriksson 2008). In the case of Rosaceae, recruitment of animals that frequently forage in forest clearings helps the plant to jump between colonization sites.

Large mammals, collectively, represent highly effective seed dispersers, due to their ability to disperse large seeds and high abundances of seeds (Tiffney and Mazer 1995; Escribano-Avila et al. 2014; Jara‐Guerrero et al. 2018). Seed dispersal mutualism usually relies on guilds of animals, what Tiffney (2004) refers to as diffuse coevolution (Janzen and Martin 1982; Wenny 2001). When the dispersers in a guild are lost, there can be direct evolutionary consequences on the plant communities that relied on them. To take one example, a recent study by Onstein et al. (2018) demonstrated both a decrease in size and an increased rate of extinction in megafruits in the Arecaceae clade throughout the Holocene. Other studies have demonstrated that the loss of megafaunal dispersers results in a loss of seed dispersal and subsequently extinction or fragmentation of large-fruiting plant populations (Galetti et al. 2006, 2018; Eriksson 2008; Malhi et al. 2016; Pires et al. 2018). Seed-dispersal studies consistently illustrate that perissodactyla (including rhinoceroses and tapirs) are far more likely to disperse large seeds and consume sugary fruits than true ruminants, notably artiodactyla (including cattle, deer, and their relatives), and are more readily featured in zoochory studies (Nathan et al. 2008; O’Farrill et al. 2013; Jara‐Guerrero et al. 2018). Extensive research has also gone into the study of proboscidean seed dispersal rates (Kitamura et al. 2007; Campos-Arceiz et al. 2008; Harich et al. 2016; McConkey et al. 2018).

## *Malus* Radiation and Diversification

While Pleistocene and early Holocene hominins undoubtedly dispersed wild apples, large-fruiting apple populations are unevenly situated across Eurasia, with concentrations in Central Europe, in areas such as the Rhine Valley and around the Caucasus, and in northern Central Asia, in the Tian Shan and Pamir Mountains. Meanwhile, endemic large-fruiting species such as *M. tschonoskii* and *M. floribunda* have restricted ranges in East Asia, as far east as Japan. These patches of mid-latitude distribution reflect the remnants left by the late Pleistocene ice sheets and permafrost, suggesting that the large-fruiting species of *Malus* have seen limited mobility over the past 20,000 years (Spengler 2019). Richards et al. (2009) noted that prior to the Last Glacial Maximum (ca. 20,000 years ago) there was likely a much larger ancestral population covering the range of all of these now-isolated populations. The main progenitor for the domesticated apple comes from a restricted population in a few river valleys in the western Tian Shan Mountains of southeastern Kazakhstan (Fig. 1) (Harris et al. 2002; Velasco et al. 2010; Cornille et al. 2014). Today heavy herd animal grazing has restricted the trees to steep slopes (between 800 and 1500 m above sea level); with low rates of seed dispersal in the current population, their range has dwindled considerably throughout the Holocene. The trees largely propagate today through root shoots and cloning, with most of their prolific generations failing to disperse and energetically costly fruits rotting below the parent trees (Omasheva et al. 2015; Duan et al. 2017; Spengler 2019). The production of large sugary fruits that, on the modern Eurasian landscape, fail to disperse would be highly maladaptive without human intervention today.

Genetic studies show that the pome-fruiting branch of the family diverged in the late Miocene, ca. nine million years ago (Töpel et al. 2012). Most of the morphological change in fruit structure seems to be tied to hybridization and polyploidy (Xiang et al. 2017). A whole genome duplication appears to be shared by all *Pyrus* and *Malus* species (Wu et al. 2013) and the formation of the Maloideae subfamily likely resulted from the hybridization of ancestral members of the Spiraeoideae and Amygdaloideae subfamilies (Dickinson et al. 2007; Xiang et al. 2017). Fossils of Rosaceae-type leaves and flowers have been recovered dating back to the early Eocene (Evans and Campbell 2002; Xiang et al. 2017), and fossil and genetic evidence suggest large-fruiting forms evolved well before the Pleistocene, starting in the late Miocene (Jongmans 1915; Reid and Reid 1915; Mai 1995; Gümbel and Mai 2004; DeVore and Pigg 2007). Some fossil studies specifically claim to identify large-fruiting *Pyrus* in late Miocene and Pliocene deposits from Northern Europe (Mädler 1939; Szafer 1947). Kvaček et al. (2020) note leaves from several different Maloideae species, including *Malus* remains from Pliocene deposits in central Germany.

The wild progenitor of the peach is regarded as once being widely distributed through northern China but is now extinct (Fedorov et al. 1971; Lu and Bartholomew 2003). Similarly, several large-fruiting species of *Prunus* exist across Eurasia, predominantly in East Asia. In general, the larger the fruit, the more restricted the modern range, and many species reproduce largely asexually—a few, such as *P. mira*, have lifespans of up to a millennium. Like large-fruiting Maloideae in Europe, *Prunus* in Asia have lost their ability to colonize new territory and have constrained gene flow (Spengler 2019). All these population features, shared across large-fruiting Maloideae and Prunoideae, suggest that they lost their ability to disperse seeds before the Holocene. Evolutionary genomics of the peach and almond suggest that the two lineages split around eight million years ago, likely in response to geological uplift of the Himalaya and climate change (Velasco et al. 2016). Assuming both lineages shared a large-seeded fleshy fruiting ancestor, then it would push large fruits in the *Prunus* clade back to the late Miocene as well. Yu et al. (2018) argue for a more recent origin of large-fruiting varieties of wild peaches, suggesting that they diverged from a shared ancestral relative with the almonds only 4.99 Ma. They argue for a shared ancestor that did not have a thick fleshy endocarp, and therefore suggest that fleshy fruiting forms evolved in southwest China during the terminal Miocene or early Pliocene. They also support Su et al.’s (2015) hypothesis of large primate dispersers (notably *Gigantopithecus*) driving evolution of large fruits. Velasco et al. (2016) point out a deep time depth for fruit divergence. The ancient origins of large-fruiting *Prunus* trees are further supported by fossils of *P. kunmingensis* from Yunnan, China, dating to the Pliocene/Pleistocene boundary 2.6 million years ago (Su et al. 2015).

## Hominids of Miocene Eurasia

About 19 million years ago, the “*Gomphotherium* land bridge” formed between Europe and Africa (Fortelius 2015). Catarrhine primates were among the taxa that dispersed into Eurasia at this time. The oldest fossil hominids in Eurasia, the griphopiths, descend from an African dispersal, had thick dental enamel, and were likely frugivorous “hard-object” feeders (Heizmann and Begun 2001; King 2001). Roughly contemporaneously, another clade of frugivorous primates, the Pliopithecoidea, make their first appearance in Eurasia. Pliopithecoids diversified into three recognized clades (pliopithecids, dionysopithecids, and crouzelids) with distinctive dental morphologies; almost all are considered to have been largely frugivorous (Ungar and Kay 1995; Kay and Ungar 1997; Deane et al. 2013). By about 12.5 Ma the thickly enameled hominids of Europe are replaced by the dryopithecins, a diverse group mainly comprised of more thinly enameled apes (Begun et al. 2012). By about 9.5 Ma most dryopithecins in Europe are extinct, but another group, best represented by the large, thickly enameled frugivorous ape *Ouranopithecus*, appears in northern Greece. Other thickly enameled apes persist in the Balkans and Anatolia until about 7.2 Ma. Finally, an ape with unique dentition, strongly suggestive of a folivorous diet, *Oreopithecus*, appears at about 8.3 Ma in Italy (Ungar and Kay 1995; Hammond et al. 2020). Most of the fossil apes from Europe and Western Asia are associated with forested ecological settings, the exceptions being the Balkan/Anatolian apes, which are associated with more open settings.

During the middle to late Miocene, these primates flourished from the area of modern Barcelona to Georgia (Begun et al. 2012) and were associated with the expansion of subtropical forests under increased atmospheric CO_2_ and high temperatures (Hamon et al. 2012; Bouchal et al. 2018). The period after the mid-Miocene Climatic Optimum (ca. 14 million years ago) was characterized by a significant global cooling (Herbert et al. 2016) associated with increasing aridity and seasonality and resulting in a vegetation shift across Europe, as forests changed from humid subtropical and evergreen to temperate, deciduous, and seasonal (Mai 1995; Agustı 2007; Denk et al. 2018). The thinly enameled dryopithecins, ranging from about 12.5 to 10 Ma, lived during the early stages of this global cooling and are associated with persistent humid forest conditions. By 9.5 Ma the cooling had progressed significantly, especially in the Balkans and Anatolia, where *Ouranopithecus* and *Graecopithecus* were found. These large, thickly enameled apes are found in drier, more open woodlands and probably consumed terrestrial as well as arboreal resources (de Bonis and Koufos 1994; Bonis and Koufos 2014; Güleç et al. 2007; Böhme et al. 2017; Koufos and de Bonis 2017).

Both the thinly enameled dryopithecins and thickly enameled *Ouranopithecus* preserve abundant morphological evidence of frugivorous diets. Dryopithecins possessed molar occlusal morphology most similar to *Pan*, large, robust upper incisors and relatively gracile masticatory apparatus, all suggestive of a diet primarily involving soft-fruit frugivory (Begun and Kordos 1997; Begun et al. 2012). This is consistent with the results of microwear, shearing quotient, and incisor curvature analysis (Ungar and Kay 1995; Kay and Ungar 1997; King 2001; Deane et al. 2013). *Rudapithecus* is also klinorynchous (ventrally deflected face), and has an anteroposteriorly elongated temporomandibular joint, both of which have been associated with an enlarging jaw gape—a possible adaptation for processing large food items with the anterior dentition (de Bonis and Koufos 1993; Terhune 2013; Gunz et al. 2020). In addition to thick enamel, *Ouranopithecus* and *Graecopithecus* share attributes of gnathic morphology with australopithecines, indicative of powerful mastication typically associated with a hard-object frugivorous diet (de Bonis and Koufos 1994; Begun and Kordos 1997; Begun et al. 2012; Böhme et al. 2017; Fuss et al. 2017).

The mid-Vallesian Crisis (ca. 9.5 Ma; a period marked by the extinction of several mammalian clades) may have caused large-scale species extinction, and both floral and faunal communities were dramatically changed (Agustı et al. 2003; Agustı 2007). Forest-dwelling fauna experienced higher extinction rates, presumably due to forest fragmentation and an increasingly mosaic landscape structure. The increased prevalence of large-bodied herbivores further illustrates a shift towards grassland and patchy deciduous forest ecology. The transition from *Pan*-like soft-fruit frugivory to hard/tough-object frugivory is probably a direct consequence of the Vallesian Crisis. However, *Oreopithecus* evolved highly derived dentitions with distinctive high-crested molars, tall cusps separated by narrow, deep basins, and a powerfully developed masticatory apparatus with robust mandibles and prominent muscle attachments, including a sagittal crest. In these attributes *Oreopithecus* resembles colobine monkeys, the most folivorous of the Old World monkeys. *Oreopithecus* is easily distinguished from colobines in many details of morphology and each have clearly converged on a superficially similar folivorous functional complex.

Asian Miocene apes, such as *Sivapithecus*,* Indopithecus*,* Khoratpithecus*, and *Lufengpithecus*, were also adapted to a range of habitats from humid forests to patchy woodland/grassland mosaics. They exhibit a range of morphological similarities seen in European Miocene apes, suggestive of frugivory ranging from soft fleshy fruits to hard/tough ones (Teaford and Walker 1984; Wu et al. 2002; Merceron et al. 2006; Nelson 2007). In addition to the evidence from gnathic morphology and microwear, additional evidence of frugivory in middle and late Miocene apes includes caries, big brains, and circumstantial evidence from genetics. Fuss et al. ( 2018) describe crown caries in the Austrian late middle Miocene (12.5 Ma) ape *Dryopithecus carinthiacus*. They relate this to the frequent exploitation of sugar-rich fruits. Crown caries also occur in the Hungarian late Miocene ape *Rudapithecus*. Caries are relatively rare in extant primates, suggesting that Miocene apes were even more committed to sugar-rich fruits than apes are today (Fuss et al. 2018). Paleobotanical evidence of food resources with cariogenic sugars at St. Stefan, Austria, where *D. carinthiacus* is found, includes plants in the *Prunus*, *Vitis*, and *Morus* clades (Fuss et al. 2018).

The pathways involved in fructose metabolism vary among primates. In hominids several genetic events have led to a unique pattern of fructose metabolism. Efficient metabolism of fructose into stored fat requires the presence of high levels of serum uric acid. In most mammals the enzyme uricase metabolizes uric acid, resulting in low serum levels and less efficient conversion into fat. In hominids, a series of mutations has suppressed the production of uricase, leading to higher levels of serum uric acid. Uncontrolled levels of uric acid in the blood stream can have negative consequences, the best known of which is gout. An overproduction of fat has many more well-known negative health consequences (Johnson et al. 2020). A second set of mutations, involving the production of the URAT 1 enzyme, enhances the regulation of serum uric acid levels and normally maintains a balance between serum uric acid, fat production, and their health consequences (Tan et al. 2016). There are many advantages to efficiently converting fructose to fat and to maintaining high serum uric acid. Metabolism of fat into energy (glycolysis) requires less oxygen than mitochondrial (ATP) energy production. Glycolysis also makes metabolic water available during periods of resource scarcity. In addition to buffering during stressful periods, fructose metabolism favors an increase in glucose levels to fuel the brain and has positive effects on immunity and blood pressure (Johnson et al. 2020). The efficient storage and metabolism of fat is crucial for organisms with high energy demands, particularly related to brain size, that are subjected to periodic (seasonal) shortages of food. There is clear evidence of seasonal ecological and dietary stress in European fossil great apes. Kelley (2008) and Skinner et al. (1995) describe patterns of dental enamel hypoplasia that are consistent with seasonal stress.

Brain function is heavily dependent on fructose-glucose metabolism. Enhanced ability to process fructose to produce glucose for brain metabolism is thought to be related to the development of enlarged brains in hominids (Johnson et al. 2020). Only three Miocene ape specimens provide anatomical evidence of adult brain size. *Ekembo nyanzae*, an early Miocene ape from Kenya, had a relative brain size similar to hylobatids and baboons; the latter is the most encephalized Old World monkey (Falk 1983; Begun and Kordos 2004). In contrast, *Rudapithecus hungaricus* had a relative brain size in the range of great apes (Begun and Kordos 2004; Gunz et al. 2020). The brain size of *Oreopithecus* has been estimated indirectly from the size of the foramen magnum as well as its relatively small braincase (Harrison 1989; Alba et al. 2001; Begun and Kordos 2004). It falls among smaller brained cercopithecoids. This is consistent with the pattern of frugivory and fructose metabolism outlined above. *Ekembo* lived after the URAT 1 mutation regulating uric acid metabolism but before the suppression of the uricase gene. This resulted in some enhancement of fructose metabolism. By the time *Rudapithecus* evolved, additional mutations had cleared the way for further enhancement of fructose metabolism that made it possible to energize a significantly enlarged brain. This larger brain in turn enhanced the ability of fossil great apes to exploit resources under challenging conditions, and the feedback loop was set in place that would lead to the immensely enlarged brains of modern humans (Begun and Kordos 2004). Estimates of the timing of the two critical mutations in hominid evolution (uricase suppression and URAT 1 metabolism) vary. The enhanced role of URAT 1 to regulate uric acid serum levels results from a series of mutations, the last of which occurred about 27 Ma (Tan et al. 2016). The mutation leading to the suppression of uricase is timed at about 17 Ma.

To summarize, researchers agree that Eurasian Miocene fossil primates inhabited a wide range of environments from swampy forests to patchy woodland/grassland mosaics. All, except *Oreopithecus*, were primarily frugivorous. Frugivory encompasses a broad range of dietary strategies and there has been little discussion of the types of fruits exploited by different Miocene apes, other than a characterization of their mechanical properties. The humid forests of Europe before about 9.5 Ma included resources such as *Ficus*, *Prunus*, and *Malus*. The open deciduous forests in the Balkans after 9.5 Ma, contained shrubby trees that produced nuts or non-fleshy fruits (Mai 1995; Denk et al. 2018). However, there is little evidence for the actual floral composition of Balkan hominid localities, in particular, the presence of fleshy fruits. Several members of the Rosaceae clade evolved during this period and their radiation further illustrates the shift towards open forb and grassland ecology. Many woody species evolved typical shrubby tree habits, characteristic of open savanna or patchy forest landscapes. Likewise, fleshy fruit production allowed for more successful directed seed dispersal and the colonization of open patches.

## Megafaunal Primate Seed Dispersal

Modern ecological studies show how effective primates are at dispersing seeds, an observation that can be extrapolated back to their frugivorous ancestors. Extensive studies across the tropics and beyond have illustrated the prevalence of seed dispersal by primates (Leighton and Leighton 1982; Lambert and Garber 1998; Cowlishaw and Dunbar 2000; Worman and Chapman 2006; Fourrier 2013; McConkey et al. 2015; Fuzessy et al. 2017). Lambert and Graber (1998) state that it “is apparent that many primate lineages exhibit dental, digestive, and/or sensory adaptations that aid in the exploitation of particular food types and that many lineages of flowering plants have evolved characteristics of fruits and seeds that facilitate seed dispersal.” Primates express more frugivorous behavior and evolutionary adaptation to frugivory than any other mammalian clade (Fleming and Kress 2011; Russo and Chapman 2011). Many primates, apes in particular, gain the majority of their caloric intake through fruit consumption (Ban et al. 2016). Primatologists estimate that 95% of tree species in tropical forests are dispersed via endozoochory (Terborgh et al. 2002). Observational studies have correlated high densities of primates, especially large-bodied species, and rich forest foraging patches, notably tree species with large fleshy fruits (Lambert and Garber 1998; Lambert 2001; Stevenson 2001). Fossil studies of Pleistocene and Holocene primates also illustrate the effective mutualism for large-fruit seed dispersal. Evidence for giant subfossil lemurs driving fruit evolution in Madagascar comes from the loss of range in all large-fruiting arboreal species following their extinction. The gradual loss of fruiting trees after the loss of the great lemurs indicates that these trees originally evolved larger fruits to attract the lemurs. Likewise, *Gigantopithecus* has been implicated in the dispersal of large-fruiting *Prunus* species in South Asia (see the following section for more details on these case studies).

## Large-Bodied Primates and Seed Dispersal

### Extant Primates

Ecologists recognize the importance of primates to tree species’ richness and abundance; in many tropical forests, fruit trees are largely limited to primate dispersers, as other large mammalian dispersers are functionally extinct (Clark et al. 2001; Koné et al. 2008). Often germination rates are extremely low without primate fruit consumption, due to heavy seed predation and fungal attack (Lambert 2001). Additionally, studies have consistently demonstrated that the loss of primate dispersal services in a forest can lead to reductions in vegetation richness and an inability for forests to regenerate (Beckman and Muller-Landau 2007; Nuñez‐Iturri and Howe 2007; Stoner et al. 2007; Wright et al. 2007; Stevenson and Aldana 2008). While some other mammalian clades, such as equids or proboscideans, opportunistically consume fruit as part of a browsing habit, primates are the only group of mammals where strict frugivory is a common practice (Turner 2001). Many studies have shown that seed dispersal is the most important ecological service provided by primates and that they increase overall biodiversity (Cowlishaw and Dunbar 2000; Russo and Chapman 2011). Primates not only consume large-seeded fruits, they also carry fruits with the seeds in them, and in some tropical forests they are one of the primary drivers of forest vegetation communities (Lambert and Garber 1998). The early fossil record suggests that primates may have diverged from other mammalian lineages as a response to obligate frugivory, notably targeting colorful avian-dispersed fruits (Sussman 1991; Reagan et al. 2001).

Large-bodied frugivorous mammals disperse a greater abundance and more diverse variety of seeds than other tropical mammals; they also spread seeds of larger sizes with more offspring provisioning (Peres 2002; Wotton & Kelly 2011). Fossil evidence illustrates that there is a correlation between bigger seeds and zoochoric dispersal (Tiffney 2004; Eriksson 2008). Larger animals have longer gut passages and, therefore, can disperse seeds to far-flung colonization locations (Ruxton and Schaefer 2012; Wotton and Kelly 2012). Several studies have demonstrated high post-digestion germination rates and extensive dispersal distances in orangutans (*Pongo abelii* and *P. pygmaeus*), chimpanzees (*Pan troglodytes*; Idani 1986; Wrangham et al. 1994; Gross-Camp and Kaplin 2005), bonobos (*Pan paniscus*), and gorillas (*Gorilla gorilla*). The seeds dispersed by chimpanzees and gorillas ranged in size from 0.1 to 2.7 cm, and seeds larger than 2.0 cm were readily dispersed over great distances (Wrangham et al. 1994). Some studies show that hundreds of seeds can be dispersed over great distances daily (Lambert 1999).

Some primate studies go so far as to imply social decision-making and reasoning in relation to access to feeding patches (Leighton and Leighton 1982), accounting for complex weather and temperature variables when choosing fruit foraging routes (Janmaat et al. 2006), and access to higher quality fruits as a preference to fruit abundance (Ban et al. 2014, 2016). Nonhuman great apes have proven to be particularly effective seed dispersers and actively select fruits based on high sugar concentration (Fuzessy et al. 2016, 2017). Many large mammals have coevolved seed-dispersal-based mutualism with large-fruiting trees, but primates are particularly successful at driving the evolution of larger fruits (Sussman et al. 2013). Primates essentially create orchards of primate-dispersed fruit trees, by consuming and carrying seeds to ideal growing sites (Lambert and Garber 1998; McConkey and Brockelman 2011; McConkey et al. 2014, 2015). Rindos (2013, p. 132) refers to these monkey hot spot forests as “monkey gardens,” noting that *Canarium* trees in the tropics tend to be central points for primate activity. Many other studies of primate-planted fruit forests show similar results (Hladik and Hladik 1967; Gartlan 1968; Glander 1975; Van der Pijl 1982).

It is difficult to ascertain what the diet of European late Miocene primates might have looked like, given that there are few extant primates that experience significant seasonality and because we lack detailed data on food sources available to them. The Japanese macaque (*Macaca fuscata*) may serve as a rough case study for at least some of the ecologies that European apes lived in, given their existence in deciduous forests that are snow-covered for a portion of the year. This level of seasonality is probably more extreme than what most European late Miocene apes dealt with; nonetheless, seasonal fruit availability would have been key to their survival. Extensive observational studies of semi-captive (in confined preserves) macaques illustrate the seasonal importance of fruits in their diet and the year-round importance of plants (Jaman et al. 2010). Similar observations were made of macaques in the Yakushima Forest (Maruhashi 1980; Hill 1997), and in a larger study spanning the range of the species, which also found variable diets based on ecological constraints (Tsuji et al. 2015). While large-fruiting Rosaceae were not among the arboreal species observed in any of these primate preserves, the macaques were important seed dispersers of fruits between 4 and 16 mm in diameter, but they also consumed smaller fruits (Noma and Yumoto 1997). Seasonally intense consumption of fruits is common in other large primate clades as well, including chimpanzees that occupy mixed ecological settings that include more open savanna landscapes. Observational studies of Bornean orangutans (*Pongo pygmaeus* ssp. *morio*) of the Danum Valley, Sabah, Malaysia, show that they will intensively consume fruits of *Dipterocarpus* during masting periods and regularly consume moderate amounts of continually fruiting *Ficus* and *Spatholobus* during periods between (Kanamori et al. 2010).

### Extinct Megafaunal Primates

Beyond a review of the hominids of Miocene Europe, another way to probe the likelihood that Miocene apes were major fruit seed dispersers is to explore what happened to fruit distributions in other cases where megafaunal primates went extinct. As an example, in South and Southeast Asia, large-bodied primates may have been a disperser of large fruits in the *Prunus* spp. clade, notably the extinct *Prunus kunmingensis* (Su et al. 2015); *Indopithecus*, at 6.5 million years ago, may or may not be related to *Gigantopithecus*, the latter mostly known from fossils between about 1 million and 300,000 years ago. *Gigantopithecus*, mostly known from China, may have also been present in Vietnam, Thailand, and Indonesia (Bocherens et al. 2017). The onset of the Pleistocene and expansion of savannas further south, eliminating the arboreal food sources, may have contributed to the eventual extinction of the clade. Reconstructions of the ecological habitat of this massive ape suggest that it occupied warm-temperate to subtropical climates, in mixed deciduous and evergreen broad-leaved forests (Jin et al. 2008; Li et al. 2014). Paleontological studies suggest that these apes shared the forested landscape with a range of other seed-dispersing primates, including *Macaca* (several species), *Rhinopithecus*, *Pygathrix*, *Trachypithecus*, *Nomascus*, and *Pongo* (Takai et al. 2014). Studies of their dental morphology and phytoliths in dental calculus suggest a largely plant-based diet, with fruits, nuts, roots, and possibly bamboo shoots (McKee et al. 2015). Stable isotope analysis of enamel in *Gigantopithecus* indicates that it was a forest dweller with a diet most like that of *Pongo* (Zhao et al. 2011; Qu et al. 2014). *Gigantopithecus* also has a relatively high frequency of carious lesions, both crown and marginal, ranging from 9.8 to 19.5% of teeth in three different samples (Han and Zhao 2002; Wang 2009). This is significantly higher than in any other sample of extant or extinct hominoids and suggests a diet that included sugary carbohydrates, notably fruits, consistent with other lines of evidence (Ciochon et al. 1990; Zhao et al. 2011; Nelson 2014; Qu et al. 2014; Bocherens et al. 2017; Zhang & Harrison 2017). Other late Pleistocene megafaunal opportunistic fruit-eating seed dispersers of Asia included the straight-tusked elephant (*Palaeoloxodon* spp.), boar (*Sus* spp.), and bears (e.g., *Ursus tibethanus*,* Helarctos malayanus*, and *Melursus ursinus*)—collectively suggesting that forests were rich in fruiting trees.

Another informative case of extinct megafaunal primates serving as the primary seed dispersers for trees with megafruits comes from the giant lemurs of Madagascar (e.g., *Megaladapis edwardsi*). Paleoprimatologists estimate that as many as ten species of lemur went extinct as recently as two millennia ago; this recent extinction allows scholars to study the effects of the loss of seed-dispersal services (Burney et al. 2004; Crowley et al. 2011). While two of these extinct species have specifically been identified as seed dispersers, *Archaeolemur majori* and *Pachylemur insignis* (Godfrey et al. 2008), several others have been identified as large-bodied fruit eaters (Crowley et al. 2011). At least eleven genera of trees with large fleshy fruits exist in southern Madagascar; all of which were likely reliant upon the giant lemurs. However, seed dispersal in some of these species does continue on a limited scale through extant smaller-bodied lemurs (Crowley et al. 2011). Genetic studies illustrate that many of these tree populations, without their giant carriers, are now fragmenting and becoming genetically isolated (Voigt et al. 2009), a similar pattern to what we see around the world in other megafruit trees during the Holocene.

Several paleontologists have noted that a general diversification of angiosperms, and specifically the development of larger fruits, occurred globally during the late Paleocene and early Eocene (Tiffney 2004; Sussman et al. 2013). The role of primates in driving the evolution of angiosperms, especially fruiting trees, was clearly laid out by Sussman (Sussman 1991), in his angiosperm/primate coevolution theory. His interest rested on the Eocene evolution of arboreal traits, color vision, and other features specifically derived in primates largely for the acquisition of fruits (Velasco et al. 2016; Yu et al. 2018). In this theoretical framework, the mutualistic link drove angiosperms to develop larger and sweeter fruits as an evolutionary adaptation for recruiting primate dispersers. Hence, the evolution of larger fruits in Europe during the late Miocene may represent a continuation of the trajectory towards diversification and radiation of angiosperms starting in the Eocene. However, like most seed dispersal, the bonds of mutualism appear to have been tied into a guild of species and mammals, including the fruit-focused lineages of primates and bats, and birds both radiate around this period (Tiffney 2004; Meredith et al. 2011). Valenta et al. ( 2018) have recently readdressed the question of whether primates were responsible for a suite of dispersal traits shared among arboreal tropical species.

## Color Vision and Visual Ripeness Displays in Fruits

Scholars have debated the likelihood that primates were a driving factor in the evolution of angiosperm fruit features (Sussman et al. 2013; Valenta et al. 2018). Some scholars refer to this as the “frugivory hypothesis” (Nevo et al. 2018; Onstein et al. 2020), and Sussman et al. (2013) emphasized the role of Eocene primates in their angiosperm/primate coevolution theory. Supporting this theory, increasing evidence seems to point to a suite of traits that have evolved in parallel among tropical trees in order to recruit primate (and similarly avian and chiropteran) dispersers (Janson 1983; Stevens et al. 2009; Valenta et al. 2013, 2015; Melin et al. 2014; Valenta 2014; Nevo et al. 2018). Discussions continue regarding which fruit characteristics are closely associated with primates as opposed to other seed-dispersing animals, and studies illustrate that primates have preferences towards fruits with certain colors (Valenta et al. 2016, 2018; Valenta and Chapman 2018). Many of the traits associated with primate-dispersed fruits would have attracted a larger guild of species, including many now-extinct megafaunal mammals, making tight coevolutionary linkages unlikely. Nonetheless, as the only mammals with trichromatic color vision, the visual displays of ripeness that most catarrhine primate-dispersed fruits utilize for signaling are a strong contender for a clade-specific recruitment feature. This view is further supported by the fact that fruits that are more closely tied with non-primate megafaunal dispersers tend to remain green, yellow, or brown at ripeness. Additionally, a recent study that mapped the distribution of bright-fruiting Arecaceae and primates with color vision noted a strongly linked relationship (Onstein et al. 2020). The authors of that study suggest that the availability of palm fruits resulted in a coevolutionary relationship between the species leading to the dynamics of primate color vision systems and palm fruit colors. They argue that the rapid radiation of primates with color vision and brightly colored palm species in Africa ten million years ago are correlated.

Avian-dispersed plants, such as the rose, have fruits that turn red when fully ripe to signal birds. Birds are attracted to bright colors, notably red, which is why so many avian-dispersed berries are red, for example, *Rubus*,* Sorbus*,* Vaccinium*,* Viburnum,* and *Rosa.* It is reasonable to assume that the red color of a ripe apple is a plesiomorphic trait given that Rosaceae fruits of the early Miocene were likely avian dispersed. However, its persistence as a trait or possible derived presence in *Malus* could be linked to disperser recruitment. Red fruits, as in wild *Malus* spp., are rare among large fruits. Most plants that relied on now-extinct megafauna that were color blind and utilized an olfactory foraging system have fruits with high sugar or oil content, and are green, yellow, or brown when ripe. “Contrary to fruit assemblages from different communities, the range of fruit colors of megafaunal species is very restricted” (Guimarães et al. 2008).

Most mammals have dichromatic vision; scholars have theorized that this basal trait is a relic of a mammalian bottleneck during the K-Pg extinction event at the Cretaceous–Paleogene boundary around 65 million years ago (Wu et al. 2017). If small subterranean and nocturnal mammals were the only clades to survive the mass extinction, then all resulting lineages would have dichromatic vision, which is advantageous under low-light conditions. Trichromatic vision evolved independently in several primate lineages; notably the great apes and howler monkeys as well as females of certain species of New World monkeys (Rowe 2018). These primate lineages likely coevolved with fruiting tree species that used bright colors to signal ripeness of fruits in order to attract frugivorous birds. Having the ability to identify red ripe fruits from a distance gave a selective advantage to both the tree and the primate, supporting the idea of a coevolutionary linkage between angiosperms and primates.

Olfactory foraging is the basal state for most mammals; however, in Old World primates, especially the apes, olfactory abilities have been de-emphasized as trichromatic color vision and better depth perception developed (Fobes 1982; Osorio and Vorobyev 1996). Primates are the only eutherian mammals with full trichromatic vision (Nevo and Heymann 2015). The unique quality of color vision in Old World primates and howler monkeys is linked to several types of cone photopigments in the eye and special neural processors (Leonhardt et al. 2009; Nevo and Heymann 2015). The loss of scent perception in favor of visual abilities is usually thought to be a major component in the evolution of the hominin lineage (Smith 1927). Studies of primates have illustrated the importance of visual cues in foraging and food acquisition, specifically demonstrating that in many primate lines olfactory-guided long-distance food acquisition is challenging (Leonhardt et al. 2009; Rushmore et al. 2012; Nevo and Heymann 2015). Interestingly, nocturnal primates tend to rely more on scent tracking, and howler monkeys (*Alouatta* spp.), the only New World primates (platyrrhines) with color vision, have reduced olfactory perception as well (Leonhardt et al. 2009; Nevo and Heymann 2015). Genetic studies of apes, Old World monkeys, and howler monkeys have shown that they possess a significantly higher proportion of olfactory pseudogenes than other New World monkeys (Gilad et al. 2004). Geneticists have linked the pseudogenes to a degradation of the olfactory receptors and an increased reliance on visual cues (Dominy and Lucas 2001). Studies suggest that Old World monkeys developed color vision 23 million years ago (Yokoyama and Yokoyama 1989), which would support the model that they developed this trait in order to obtain angiosperm fruits that were intended for birds. There is also reason to believe that the loss of olfactory reception is tied in to the loss of pheromone signaling in primates (Gilad et al. 2004).

## Megafaunal Monkeys and Megafloral Roses

**Fig. 3:**
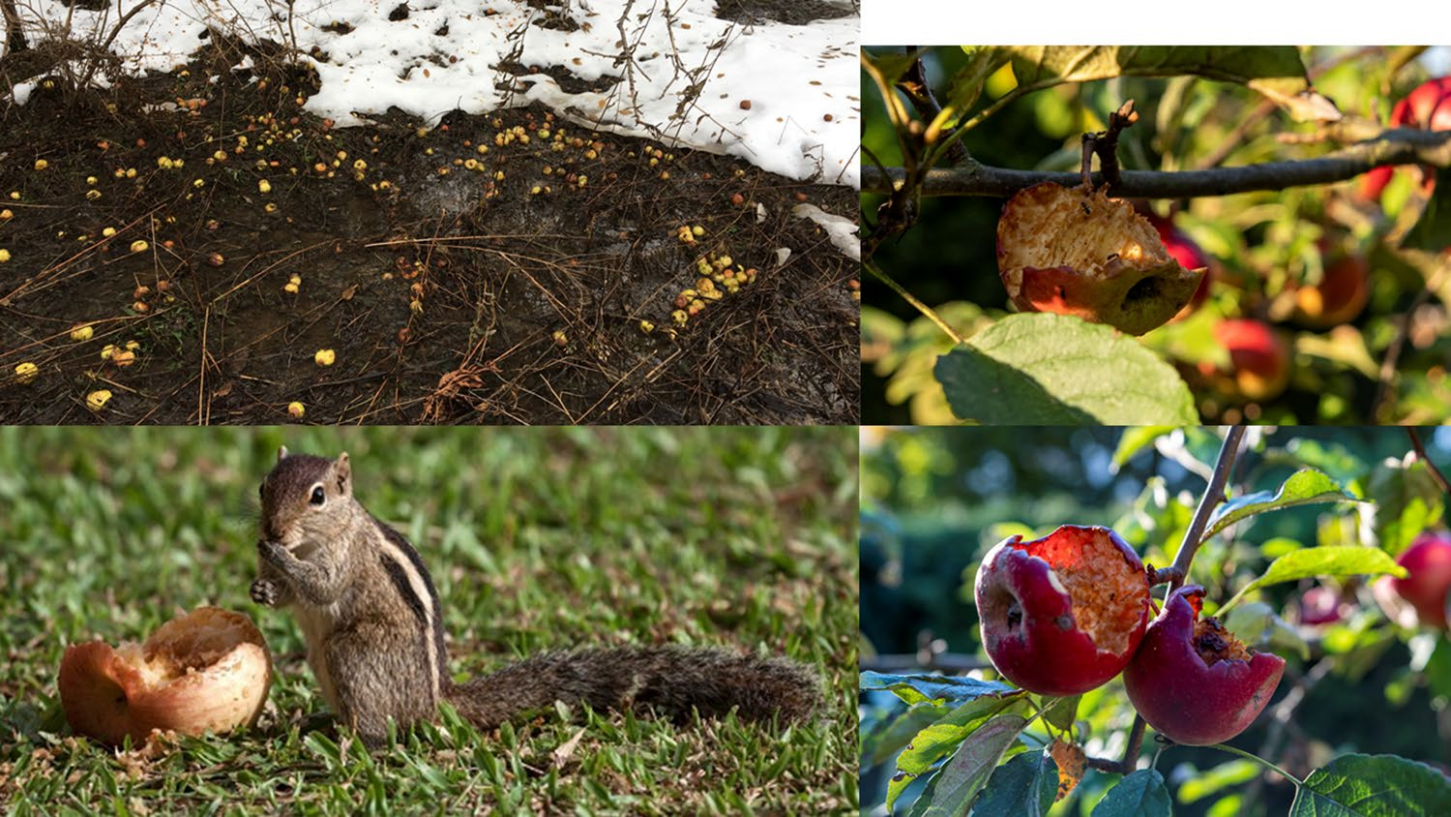
Modern observations of apple consumption and seed dispersal suggest that birds and rodents either avoid the seeds or destroy them through seed predation. Likewise, ungulate grazers largely avoid or only selectively consume the fruits. Counterclockwise from bottom left: (A) a squirrel gnawing through an apple in order to consume the seeds; (B) two images of partially consumed fruit—birds ate the fruit but failed to disperse the seeds; and (C) apples lying in a cattle field and avoided by the grazers

Many of the megafaunal-dispersed fruits that survived into the first half of the Holocene have become important components in the human diet and benefit from anthropogenic seed-dispersal services (Levis et al. 2017, 2018; van Zonneveld et al. 2018). Tropical “megafruits” have maintained their gene flow regimes, due to the continual presence of at least some megafauna, including humans and bears, certain large clasping birds such as parrots, and bats. However, in temperate zones, most members of the former disperser guilds are now extinct and much of the extant megafauna is composed of grazing animals. The lack of *Gomphotheridae*, Xenarthra, and lower densities of *Mammutidae* in Europe during the Pleistocene as opposed to the Americas further complicates the question of what species served as seed dispersers. Ruminant grazers and animals with multichambered stomachs tend to avoid sugary fruits, which ferment in the gut and cause high rates of methane production; additionally, their restricted caecum blocks the rapid processing of large seeds (Fig. 3). While medium-sized omnivores, notably boars, bears, and humans, opportunistically disperse these species, most megafruiting trees in temperate zones and in many tropical regions are currently endangered. While the paleo-distribution ranges in these species require further study, they appear to have slowly lost range throughout the Holocene (Galetti et al. 2018; Pires et al. 2018; Spengler 2019; Onstein et al. 2020).

The rose hip is small and red, in most cases small enough for birds, which do not masticate their food. The apple is clustered with other mega-rosaceous fruits, including the pear (*Pyrus*) and quince (*Cydonia*). These mega-fruits evolved to attract a seed disperser much larger than a bird; therefore, we can think of an apple tree as a mega-floral rose bush. Looking at the temperate forests of Eurasia, there are fewer potential seed dispersers than in the tropical belt. Likewise, many animals that may seem like candidates for a dispersal guild either ignore or only selectively consume sugary fruits, such as ruminants (see Fig. 3, illustrating feral apples rotting in a cow pasture). Additionally, many small mammals serve as seed predators and birds fail to disperse the seeds (Fig. 3). Hominids were the only megafaunal dispersers in late Miocene Eurasia with trichromatic vision, allowing them to observe, and select for, the evolved red color of ripe *Malus* spp. fruits. They were also the only large arboreal animals. Ripe apples often remain on the tree even after the leaves fall due to a lack of function in pedicle abscission zones, a typical adaptation for megafaunal-dispersed fruits that helps them avoid rodent seed predation.

Comprehensive studies of the fossil record of Miocene Eurasia suggest that fleshy-fruit-based endozoochory was rare overall on a landscape dominated by grazers and open woodlands (Fortelius et al. 2006). However, simultaneously, fleshy-fruiting members of the Rosaceae clade radiated and diversified. At least two independent lineages, Maloideae and Prunoideae, developed larger fruits in parallel, simultaneously evolving higher sugar concentrations. Seed dispersal is important in maintaining diversity and genetic connectivity within and across populations (Nathan and Muller-Landau 2000; Jara-Guerrero et al. 2018). Seed dispersal is also essential for colonization, especially in arboreal species (Escribano‐Avila et al. 2014). Therefore, identifying the members of a dispersal guild can inform scholars about the evolutionary processes at play on the largely savanna or patchy forest landscape of Eurasia during the late Miocene. While this landscape would have contained high densities of animal species, many of them, such as small mammals and birds, would have failed to disperse seeds, either due to seed predation or seed avoiding (Fig. 3). Additionally, grazing animals mostly avoid fleshy fruits, and in the case of ruminant digesters would have destroyed seeds of large Maloideae species. Although primates are not the only potential species that may have dispersed rosaceous seeds in Eurasia during the late Micocene, they were likely a prominent disperser in the guild (Table 1).

**Table 1:** Supporting evidence for late Miocene Apes and Rosaceous tree mutualism

Red displays of ripeness in fruits tend to attract either birds or primates, both of which possess color vision
The fruits are too large for an avian disperser, and most small or medium-bodied mammals serve as seed predators
Ruminant digesters often avoid (or consume in limited quantities) fleshy fruits with high sugar content, due to the double digestion process and grazing habit
Modern primates are some of the most effective seed dispersers in tropical forests, setting a precedent for a broader trend in the clade
The only major case study for a temperate zone primate in a seasonal forest, the Japanese macaques, are prominent seed dispersers.
Gnathic morphology suggests that many fossil apes in Europe were frugivorous, and few non-rosaceous fruiting trees would have been present on the landscape
Dentil carries and use wear on fossil primate teeth from Eurasia suggest a high fruit-based diet, specifically of high-sugar fruits
Other extinct mega-primates, such as *Gigantopithecus* and giant subfossil lemurs, appear to have been seed dispersers
While there were other frugivores or opportunistic browsers on the late Miocene landscape, a significant portion of the herbivores were grazers
*Malus* spp. maintain smaller seeds, possibly suggesting a disperser that had a restrictive digestive system
Hominids of the Pleistocene onward maintained a close mutualistic relationship with large-fruiting Rosaceae, and it is likely that this trend stretches back in time

## Conclusion

Most examples of endozoochoric megafruits have muted colors, often yellow, green, or brown. *Asimina triloba* of North America or related *Annona* fruits of Central and South America are good examples, with large hard seeds, soft sweet flesh, and remaining green when ripe. Additionally, many, such as the cucurbits and *Maclura pomifera*, do not possess high sugar concentrations. Wild apples contain red morphotypes, have high sugar levels, and they contain small easily crushed seeds. Fruits that were likely dispersed by elephantine or xenarthran species often contain large seeds or pits, easily mashed fruit coats, muted colors, and oily or low-sugar fruits. Smaller fruiting members of Rosaceae attract avian dispersers; however, the fruits of many Maloideae and Prunoideae are too large for frugivorous birds to swallow. It is probable that red fruits of *M. sylvestris* and *M. sieversii*, which are too large for avian dispersal, evolved to attract a megafaunal disperser that possessed trichromaticism, likely one that cannot swallow large pits. Additionally, there were fewer frugivores on the pre-Holocene landscape of Europe than in the Americas, where gomphotheres and other proboscideans, and xenarthrans likely drove the evolution of larger fruits. Ruminant dispersers and other grazing animals avoid sweet fleshy fruits and tend to be destructive of larger seeds.

A fleshy fruit dispersal guild in late Miocene Europe likely included bear, rhinoceros, proboscideans, equids, and suids, all of which would have selected for larger fruiting hybrid examples of *Malus* trees. Large primates were prominent on the European landscape during the Miocene. Fossil evidence seems to suggest that these primates evolved new traits to adapt to ecological changes during the late Miocene, including dental features that suggest specialized frugivory. The limited availability of other large-fruiting trees in late Miocene Europe points to a strong focus on Rosaceae. Similarly, studies of fruit consumption among both extant and extinct apes further supports the present hypothesis. Identifying the exact drivers of evolution is always difficult, and organisms often evolve in a complex milieu of selective forces, many of which are complementary. Likewise, seed dispersal often relies on guilds rather than a specific species; however, humans appear to have been the main dispersers for *M. sylvestris* in Europe since at least the Mesolithic and of *M. sieversii* and *M. orientalis* during the past few millennia. Given the close relationship between people and the apple over the past few hundred thousand years, as evidenced in the archaeobotanical record, it is reasonable to assume a deeper time depth for this mutualism. Further paleontological research, especially targeting rare fleshy fruit fossils, may validate or refute the theories that we present here, but in either case, it is clear that collaborations between domestication scholars and evolutionary ecologists can prove to be fruitful. Ultimately, this study demonstrates that the coevolutionary process of human-driven evolution that we colloquially call “domestication” has deeper roots in our hominin lineage.
